# Properties of Microplasma Coating on AZ91 Magnesium Alloy Prepared from Electrolyte with and without the Borax Addition

**DOI:** 10.3390/ma15041354

**Published:** 2022-02-12

**Authors:** Magdalena Bisztyga-Szklarz, Ewa Rząd, Łukasz Boroń, Piotr Klimczyk, Tomasz Polczyk, Aneta Łętocha, Maria Rajska, Marek Hebda, Piotr Długosz

**Affiliations:** 1 Łukasiewicz Research Network-Krakow Institute of Technology, Zakopiańska 73, 30-418 Kraków, Poland; magdalena.bisztyga@kit.lukasiewicz.gov.pl (M.B.-S.); ewa.rzad@kit.lukasiewicz.gov.pl (E.R.); lukasz.boron@kit.lukasiewicz.gov.pl (Ł.B.); piotr.klimczyk@kit.lukasiewicz.gov.pl (P.K.); tomasz.polczyk@kit.lukasiewicz.gov.pl (T.P.); aneta.letocha@kit.lukasiewicz.gov.pl (A.Ł.); 2 Faculty of Materials Science and Ceramics, AGH University of Science and Technology, A. Mickiewicza 30 Av., 30-059 Kraków, Poland; rajska@agh.edu.pl; 3 Faculty of Materials Engineering and Physics, Cracow University of Technology, Warszawska 24, 31-155 Kraków, Poland

**Keywords:** corrosion behaviour, protective coatings, plasma electrolyte oxidation, magnesium alloys, coating electrodeposition, abrasive wear

## Abstract

Magnesium alloys, due to their unique properties, low density and high strength properties, are becoming more frequently used in industrial applications. However, a limitation of their use may be the need to ensure high abrasive wear resistance and corrosion resistance. Therefore, magnesium alloys are often protected by applying protective coatings. The paper presents the influence of the modification of the electrolyte composition, with or without the addition of borax, on the morphology (observed by SEM method) and phase composition (analyzed by EDS and XRD) of the formed layers on the AZ91 magnesium alloy, and their abrasive wear (determined with Ball-on-Disc method) and corrosion resistance (evaluated using the immersion method and by electrochemical tests), especially in chloride solutions. It has been clearly demonstrated that the modification of the electrolyte composition significantly impacts the final properties of the protective coatings on the AZ91 alloy formed by the plasma electrolytic oxidation (PEO) process. On the basis of the results, it was found that the new type of PEO coatings with the borax addition, compared to base PEO coatings, showed significantly higher abrasion resistance and an order of magnitude lower corrosion rate.

## 1. Introduction

Magnesium and its alloys, due to their density, are classified into the group of so-called “*light metals*”. Moreover, their specific properties, such as, high mechanical properties and excellent castability, made them widely used in transport and production of computers, communications and consumer electronics (3C) manufacturing in order to meet environmental protection and energy saving requirements [1,2].

Unfortunately, a certain limitation in the use of magnesium and its alloys may be its higher chemical activity compared to other commonly used non-ferrous alloys, such as aluminum. As a consequence, it exhibits lower corrosion resistance [3,4]. Therefore, in order to be able to use magnesium alloys in engineering applications, they must first be properly secured, e.g., by applying protective coatings. Among various commercial magnesium alloys, the AZ (Mg–Al–Zn) series is one of the most widely used, due to its excellent mechanical properties [5,6]. The presence of an aluminum additive in the composition of the alloy, preferably up to 10%, not only significantly increases the tensile strength through the formation of the Mg_17_Al_12_ intermetallic phase, but also improves its corrosion resistance. The key factor, however, is the volumetric share of the phase formed and the place and distribution of its crystallization [7,8]. When the *β*-phase (Mg_17_Al_12_) possesses a continuous nature and is finely divided, the corrosion resistance increases [9]. On the other hand, when Mg_17_Al_12_ formed as intergranular precipitates, microgalvanic cathodes occur, decreasing the corrosion resistance of magnesium alloys [10]. Zinc is the second component, after Al, that holds the greatest influence over the properties of magnesium alloys. As with Al, it increases the strength and yield strength of magnesium alloys, but only in the presence of Al and Mn. Too much additional zinc (over 5%) causes hot brittleness and the formation of microporosity in the alloy, whereas manganese, which is the basic component found in almost all magnesium alloys, does not significantly affect the mechanical properties, but increases the corrosion resistance of magnesium alloys in saltwater, because it limits the negative influence of iron, which is the cause of corrosion [11]. However, Mg can induce the precipitation of secondary Al-Mn and/or β-Mg_17_Al_12_ particles, distributed discontinuously in structure and, thus, due to their relatively high cathodic potential, contribute to decreasing the corrosion resistance of the material [12].

In order to protect the surface of magnesium alloys and, thus, enable their widespread applications, regardless of the environmental conditions in which they would be used, in recent years, different coating technologies have been developed [13,14]. The most common methods involve the formation of stable oxide layers on their surface, followed by the application of paints, enamels, or other top coatings [15]. Coatings obtained by the plasma electrolytic oxidation process (PEO) are characterized by good corrosion and abrasion resistance [16], and the possibility of their production on various substrates [17]. However, despite the significant progress in the synthesis of this type of coating, research is still underway on: the dependence of the influence of the composition and microstructure of the substrates [18], the electrolyte used and the process parameters [19], on the formation phenomenon and the final properties of the obtained coating [20]. Moreover, this method is environmentally friendly, and the procedure is usually performed in solutions that do not contain toxic components [21].

One of the main advantages of applying the PEO coating to the surface of the material is the increase of their resistance to corrosion and abrasion [22]. However, in order to be able to use them as a permanent protective barrier against corrosion, the chemical composition of the electrolyte should be appropriately modified [23,24], and additional sealing, e.g., by applying a layer of protective paint, may be necessary. The composition of the electrolyte and its concentration are key parameters that have a significant impact on the final properties of the produced coatings. Nowadays, it is also important that the electrolytes used must be safe for both the environment and the health of workers. One such substance is borax (Na_2_B_4_O_7_·10H_2_O), which is widely used in various industrial applications [25,26]. Borate is closely related to the coating property, e.g., sodium borate can significantly change the thickness of the applied coating [25] or its corrosion resistance [25,27]. For example, Duan et al. [25] showed that when the electrolyte contained the simultaneous addition of borate and fluoride, the produced protective PEO coatings on AZ91 magnesium alloys were characterized by very good corrosion resistance in a 3.5% NaCl solution.

In this study, the properties of the coatings produced on the magnesium alloy AZ91 using the PEO process were investigated. The influence of the modification of the electrolyte composition, with or without the addition of borax, on the morphology and phase composition of the formed layers and their abrasive wear and corrosion resistance, especially in chloride solutions, was analyzed.

## 2. Materials and Methods

### 2.1. Preparation of Samples, Electrolytes, and PEO Coatings

Table 1 presents the chemical composition of the AZ91 magnesium alloy, which was determined with the GDS-850A LECO emission spectrometer. The obtained results were compared with the values determined for this alloy according to the PN-EN 1753: 2001 standard. The chemical composition of the magnesium alloy used was in line with the above criteria.

The AZ91 magnesium alloy (MgAl9Zn1) was melted in a resistance furnace (PTM-15/G, CZYLOK, Jastrzębie Zdrój, Poland) at a temperature of 680–700 °C using an SF6 protective atmosphere. Then the ingots were prepared by the coquille casting method. The PEO coatings were applied to the samples after their casting, without any prior special preparation or degreasing of their surfaces. Table 2 presents the composition of the electrolytes used and the conditions of the PEO process.

**Figure 1:**
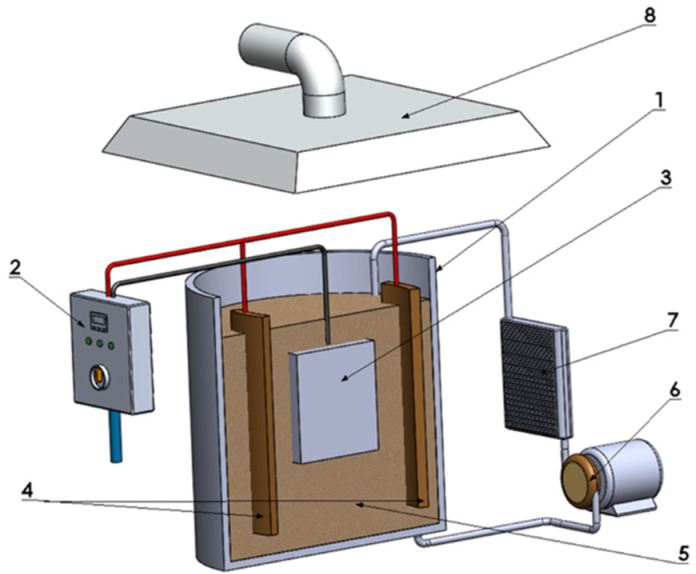
Schematic diagram of coating electrodeposition: (**1**) electrolytic tank, (**2**) high a AC power source, (**3**) sample (anode), (**4**) cathode, (**5**) electrolyte, (**6**) electrolyte pump (stirring system), (**7**) heat exchanger, (**8**) vapor extraction hood.

During the PEO process, the electrolyte temperature was controlled and kept below 20 °C. The sample was the anode and the stainless-steel container containing the electrolyte was the cathode. The diagram of the entire PEO system was presented in Figure 1. After applying the PEO protective coatings, the samples were rinsed in distilled water and then dried. The samples prepared in this way were then subjected to microstructure observations, wear and corrosion resistance analysis.

The type and the used designation of the samples were presented in Table 3.

### 2.2. Characterization of the Substrates and PEO Coatings

The surface and cross-sectional morphology, as well as the elemental composition of the PEO coatings and alloys, were examined by scanning electron microscopy (SCIOS FEI), equipped with an energy-dispersive spectrometer (EDS). X-ray diffraction (Panalytical Empyrean with PIXcel3D detector) was carried out to determine the phase composition of the coatings. The rectangular specimens were scanned in Bragg–Brentano geometry in a 2θ range of 10° to 100°, using Cu-Kα radiation. The scanning rate was 1.5°·min^−1^ with a 40 mA current and 40 kV tube voltage. The qualitative analysis was performed using HighScore Plus software (Malvern Panalytical B.V., Almelo, The Netherlands). The phase composition was identified on the basis of data contained in the International Centre for Diffraction Data (ICDD) PDF-4+ catalogue.

**Figure 2:**
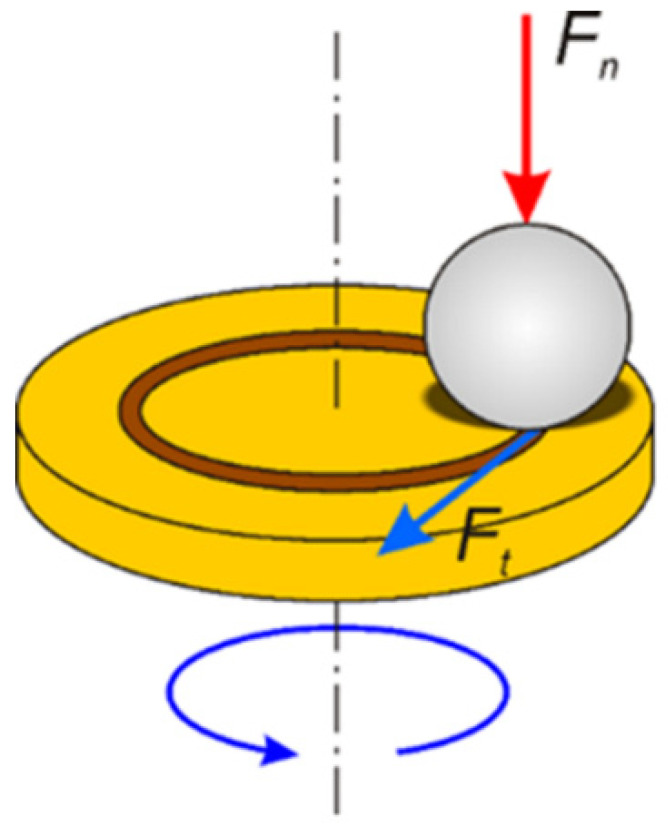
Scheme of the Ball-on-Disc arrangement.

The coefficient of friction and the wear resistance of the coatings were determined in the ball-on-disc tests, using a UMT-2MT universal mechanical tester (CETR, Campbell, CA, USA), by rotating the sample against a stationary ball (Figure 2). The loading mechanism applied a controlled load F_n_ to the ball holder. The friction force F_t_ was measured continuously during the test using the extensometer. The tests were carried out at room temperature under dry sliding conditions.

**Figure 3:**
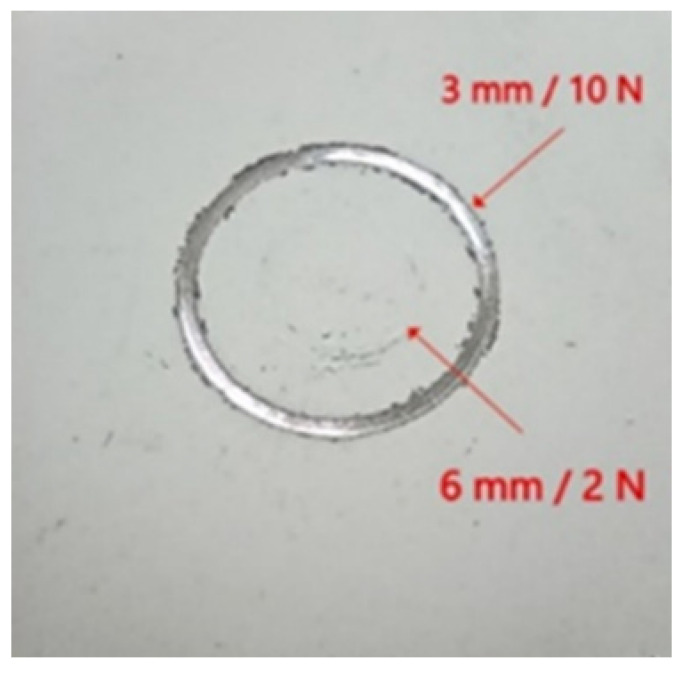
Macrophotography of specimen after preliminary trials showing the influence of ball diameter and load on the coating.

Ball diameter, load and the radius of the sliding circle were experimentally selected through preliminary analysis in such a way that the expected abrasion of the coating did not occur too early, preferably in the middle of the test (Figure 3). In the case of “too high” parameters, as was the case with the preliminary test using a ball with a diameter of 3 mm and a load of 10 N, the coating was damaged too early due to too high Hertzian contact stress. Increasing the ball diameter to 6 mm and reducing the load to 2 N resulted in decreasing of Hertzian contact stress and created conditions for a reliable comparison of the investigated coatings with each other.

Table 4 shows the selected ball-on-disc tests parameters used for all investigated coatings.

The corrosion resistance of the coatings was evaluated using the immersion method (500-h exposure) in an inert salt spray in 5% aqueous NaCl solution with pH 6.6 ÷ 7.2 at 35 °C.

To further evaluate the corrosion resistance of PEO coatings, electrochemical tests were performed. For this purpose, AUTOLAB PGSTAT302N potentiostat/galvanostat was used. In each test, a traditional three-electrode system was used, where the sample working electrode was the tested sample with surface exposed size of 1 cm^2^. The reference electrode was a chlorosilver Ag/AgCl electrode (containing 3M KCl), and a steel wire (SS316) served as the counter-electrode. The experiments were carried out in 0.1M NaCl aqueous solution. Linear sweep voltammetry (LSV) was performed at a scan rate of 0.001 V/s after an initial 1800 s exposure in solution to stabilize the open circuit potential (OCP). All measurements were made at room temperature.

## 3. Results and Discussion

### 3.1. The Coatings Morphology and Structure

**Figure 4:**
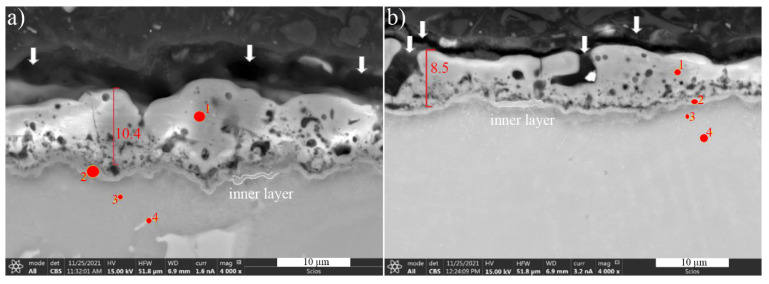
Cross-section morphology of (**a**) base PEO coating and (**b**) modified PEO coating on AZ91, observed via SEM. Arrows indicate the microporosity on the sample surface, while points 1 to 4 indicate the area of EDS analysis (Table 5).

SEM images of the surface and cross-sections of PEO coatings formed on AZ91 alloy were presented in Figure 4. Usually, after applying the PEO coating on the alloy, a two- or three-layer microstructure can be observed, with a characteristic thin barrier layer with a thickness of several hundred nanometers, formed at the substrate/coating interface [29]. Regardless of the alloy casting method and electrolyte composition, the PEO coating thicknesses were similar and ranged from 8–11 µm, with a visible thin inner layer. On this basis, it can be concluded that the coatings possessed a relatively similar growth rate. Based on the cross-sectional analysis, good cohesion of the produced coating with the substrate (magnesium alloy) was also found. On the surface of the produced coatings, it was possible to observe the uniformly distributed microporosity, characteristic for this process. It does not tend to create an interconnected network that would significantly degrade the properties of the fabricated coating. However, locally, certain microchannels may connect the substrate to the sample surface. It is important that the change of the electrolyte composition by introducing the addition of borax did not affect the amount and size of porosity and microchannels present in PEO coatings. Thus, the surface roughness was unchanged regardless of the electrolyte composition used. Larger pores locally visible on the cross-sectional of coatings are the result of discharge/spark concentration and/or evolved of gas, oxygen or hydrogen, or both, produce on the surface of the material during the PEO process. The consequence of this phenomenon was also numerous small and mutually isolated micropores visible on the cross-section just above the surface of the sample. On the other hand, the correct selection of the PEO process parameters was confirmed by the fact that the microstructure directly under the coating has not been modified in relation to the material core.

The element content (at. %) was determined from the EDS results (shown in Table 5). The analysis of PEO coatings produced on the AZ91 alloy showed that the main components are magnesium and oxygen, the same as for the base magnesium alloy. The inner layer is very thin and consists of Mg and O. This confirms that this film is the passivation layer of MgO.

**Figure 5:**
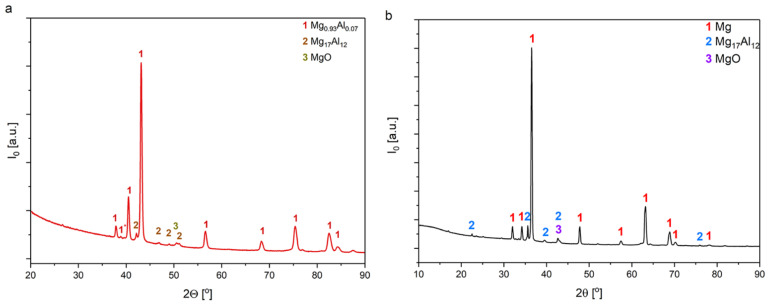
X-ray pattern of the (**a**) basic PEO coating, (**b**) modified PEO coating by the addition of borax to the electrolyte, applied to the surface of the AZ91 alloy.

Figure 5 shows the results of XRD pattern of PEO coatings, base and modified with borax addition, applied to AZ91 alloy.

The main phase identified is Mg_0.93_Al_0.07_ (card no. 04-003-5282) crystallized in a hexagonal structure. This phase appears for all samples. Furthermore, signals attributable to the Mg_17_Al_12_ (card no. 04-005-4961), and MgO (card no. 01-085-5649) phases are observed, but their signal intensities are much lower. The results suggest that the building blocks of these types of coatings closely depend on the composition of the alloy as well as the electrolyte. A consistent feature for all such coatings is the presence of a small number of oxides (MgO), which were formed according to Equations (1) and (2).

Mg^2+^ + 2OH^−^ → Mg(OH)_2_ (1)

Mg(OH)_2_ → MgO + H_2_O (2)

Moreover, slight amounts of (Mg,Al)_2_SiO_4_ are also observed, which is known by the name forsterite or olivine in mineral databases. It is a magnesium orthosilicate (salt) formed by fusion of stoichiometric amounts of magnesium and silicon oxides at 1900 °C (such a temperature may occur during the microplasma oxidation process). Mg_2_SiO_4_ was formed according to Equation (3):

2Mg+ SiO_2_ + 2H_2_O = Mg_2_SiO_4_ + 4H^+^ + 4e^−^ (3)

The presence of the Mg_17_Al_12_ phase is not unusual, especially for cast alloys of the Mg-Al system. However, the transition phase may be the result of thermal phenomena (over melting, recrystallization, and phase transformation) occurring during the plasma discharge. In the PEO processes, the magnesium substrate acts as an anode in silicate-based electrolytes; thus, the magnesium dissolution reaction (Equation 4) occurs under a strong electric field that produces magnesium ions.

Mg = Mg^2+^ + 2e^−^ (4)

The formation of oxide films on magnesium is due to the external diffusion of magnesium ions, flow from the substrate to the electrolyte, and the internal diffusion of SiO_4_^2−^, OH^−^, from the electrolyte to the substrate, at high potentials. When the concentrations of these ions at the electrode/electrolyte interface reach a critical value, film forming reactions occur.

### 3.2. Wear Resistance of the Coatings

**Figure 6:**
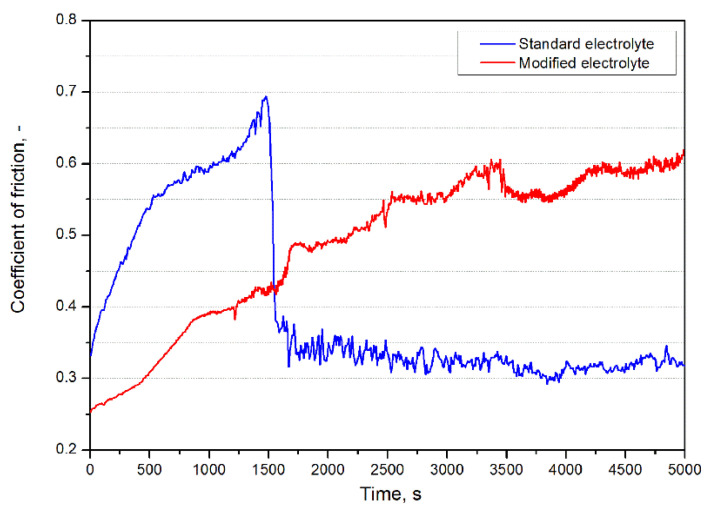
Friction coefficient measured in the Ball-On-Disc test on AZ91 magnesium alloy with coatings manufactured using standard and modified electrolytes.

The results of the ball-on-disc friction measurements on AZ91 cast alloy protected with the PEO coating was shown in Figure 6. It was observed that the coefficient of friction (COF) of an AZ91 alloy coated with the use of a standard electrolyte intensively increases in the first stage of the test, which is a typical phenomenon associated with the running-in process. Later, after about 1500 s of the test, there was a sharp decrease in COF. This phenomenon was related to the abrasive destruction of the PEO coating during the test. Destruction of the coating was also confirmed by macro-observation of the sample after the test. After the layer was broken, the COF of the AZ90 alloy was approximately constant (~0.3) until the end of the test.

On the other hand, the behaviour of the COF curve recorded for the PEO coating made on AZ91 alloy in the electrolyte containing the addition of borax also showed an increase in the initial stage, but it was not so rapid and its stabilization with the value of about 0.6 took place after ~3500 s of the analysis. Moreover, this type of PEO coating was not damaged until the end of the test, the behaviour of the COF curve was stable, and no visible signs of abrasion were observed.

### 3.3. The Corrosion Resistance of the Coatings

**Figure 7:**
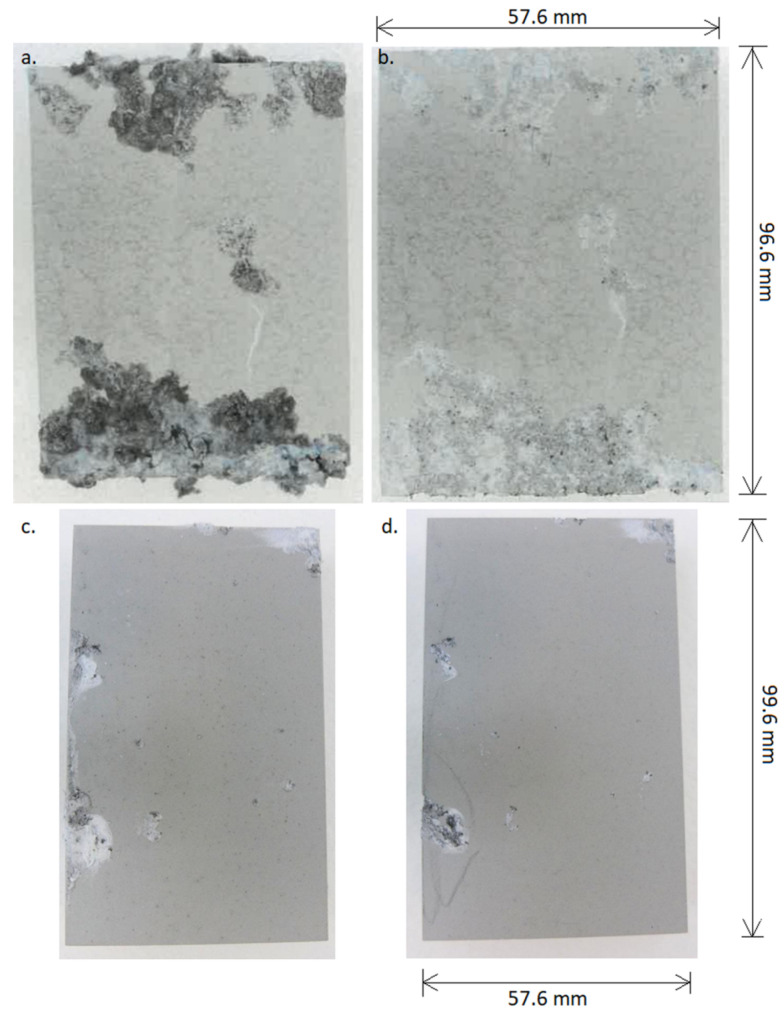
Photographs of PEO coatings on AZ91 alloy after a 500-h test in an inert salt spray (**a**) base PEO coating, (**b**) base PEO coating after removal of corrosion products, (**c**) modified PEO coating, (**d**) modified PEO coating after removal of corrosion products.

Figure 7 shows the view of the surface samples after the salt spray corrosion resistance test of the PEO coatings formed on the AZ91 magnesium alloy.

Examination of the surface of the samples after immersion in salt spray revealed surface defects and pitting, regardless of the composition of the electrolyte used in the PEO process to create the protective coating. However, for a sample covered by the base PEO process, the number and size of pits are much larger than for the sample protected when a modified PEO electrolyte was used. The coating destruction mechanism is caused by the formation of numerous new pitting corrosion centers, which grow and interconnect, mainly visible as a large dark area (hole) covering a large part of the surface on base PEO coating (Figure 7a). On the other hand, the use of a modified electrolyte with borax addition to form a PEO coating on AZ91 alloy showed a corrosion inhibitor effect (Figure 7c). On the basis of the obtained results, it can be concluded the corrosion resistance in the salt spray condition of PEO coatings formed in the electrolyte mixture with borax was much higher compared to that of PEO coatings formed in the base electrolyte.

**Figure 8:**
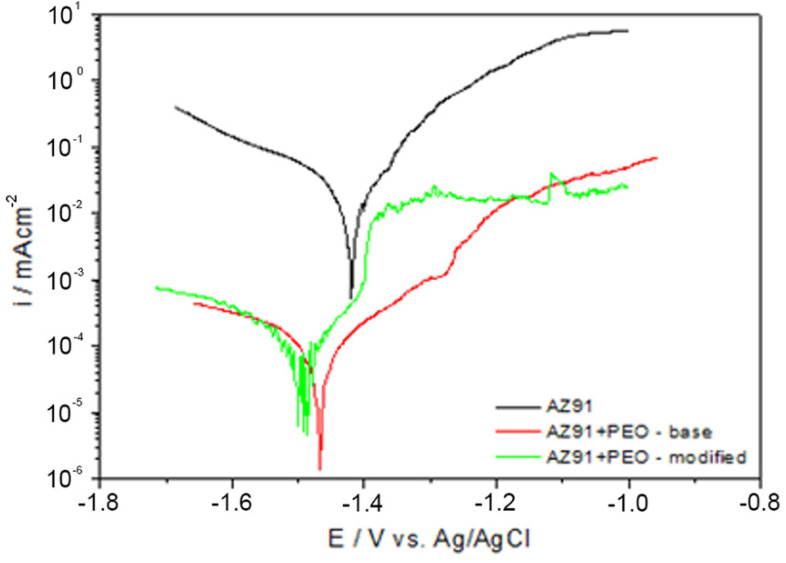
Polarisation curves of AZ91 magnesium alloy (black curve) and sample with base PEO coating (red curve), and modified PEO coating (green curve) after 30 min exposure in 0.1M NaCl.

The widespread presence of chlorides in the surrounding environment and their complexing properties and increasing the electrolyte conductivity resulted in the selection of an aqueous NaCl solution with a concentration of 0.1 mol dm^−3^ for further corrosion tests and quality assessment of the produced coatings. Figure 8 shows representative polarization curves of AZ91 alloy and samples after applying two variants of PEO protective coating. In general, it was found that the nature of all the curves is similar, however, the position of the curves recorded for the samples with PEO coatings clearly differs from the curve obtained for the AZ91 magnesium alloy. Tafel extrapolation method has been employed to determine the corrosion resistance of examined samples (Table 6).

On the basis of the recorded curves, it was found that after the application of the PEO coating to the surface of the magnesium alloy, there was a decrease of almost two orders of magnitude in the value of the cathode current density (Figure 8 red and green curves) in relation to the reference sample AZ91 (Figure 8, black curve). This effect was independent of the type of PEO coating applied. The relationship between the values of *E_corr_*, *i_corr_*, R_p_ and the corrosion rate of the tested materials clearly confirm the significant influence of PEO coatings on the protection of magnesium alloys against corrosion. The corrosion rate was reduced by two orders of magnitude. Moreover, the obtained results indicate a significant advantage of PEO coatings modified with the addition of borax, because their corrosion rate value is an order of magnitude lower compared to the base PEO coating. The recorded results are in accordance with the results obtained from the salt spray tests, wherein, for the case of the sample protected with the base PEO coating, active dissolution of the material was observed, while for the PEO coating modified with borax, the sample was attacked only in points at the discontinuity of the coating.

**Figure 9:**
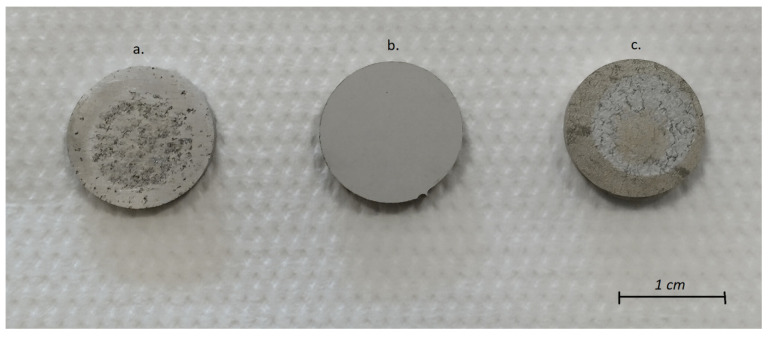
Surface photographs of (**a**) AZ91 magnesium alloy, (**b**) AZ91 magnesium alloy with modified PEO coating, and (**c**) AZ91 magnesium alloy with base PEO coating, after potentiodynamic polarization tests in 0.1M NaCl.

The surface morphology of the samples after electrochemical testing and ultrasonic cleaning (immediately after testing) was also observed in macro mode, as shown in Figure 9.

Based on the observations, it can be concluded that the most intense corrosion processes took place on the AZ91 alloy surface (Figure 9a), which is consistent with the results of electrochemical tests presented earlier. On the surface of the sample with the standard PEO coating (Figure 9c), numerous traces of corrosion are also visible, but their number and depth of pitting were smaller compared to the sample without the coating. On the other hand, the PEO coating made of the electrolyte containing the addition of borax (Figure 9b), despite the influence of the corrosive medium, remained practically undamaged, which allowed for full protection of AZ91 alloy against the influence of 0.1M NaCl.

## 4. Conclusions

Based on the presented results, it has been clearly demonstrated that the modification of the electrolyte composition significantly impacts the final properties of the PEO coatings. The coatings made on the AZ91 magnesium alloy in the electrolyte containing the addition of borax showed much greater abrasive wear resistance and corrosion resistance, especially in chloride solutions, compared to the coatings made in the solution without borax addition, and in relation to the AZ91 alloy itself. Moreover, the addition of borax to the electrolyte did not change the phenomenon of the formation and growth of the protective coating applied on the AZ91 magnesium alloy. The use of the PEO coating modified with the addition of borax changed the active dissolution of the magnesium alloy to pitting corrosion of the alloy in the places of discontinuity/porosity of the coating, which was confirmed by both visual observations and the results of the polarization test. Their corrosion rate was an order of magnitude lower than that for the base PEO coatings. Moreover, PEO coatings with the addition of borax, in contrast to the base PEO coatings, were not damaged until the end of the ball-on-disc test, and no signs of abrasion were visible on their surface. 

## Figures and Tables

**Table 1:** Chemical composition of the AZ91 magnesium alloy, and guidelines in accordance with EN 1753:2019 standard [28].

Symbol	Chemical Composition [wt%]								
	Al	Zn	Mn	Si	Fe	Cu	Ni	Ti	Mg
AZ91	8.50	0.75	0.13	0.02	0.02	0.01	0.002	0.025	Balance
EN 1753: 2019 standard	8.50 ÷ 9.50	0.30 ÷ 1.00	<0.00 ÷ 0.15	<0.30	<0.03	<0.025	<0.001	–	Balance

**Table 2:** PEO process conditions and electrolyte composition.

		Base PEO Coating	Modified PEO Coating
Electrolyte composition	NaOH (g·cm^−3^)	4	4
	Na_2_SiO_3_·5H_2_O (g·cm^−3^)	15	15
	NaF (g·cm^−3^)	-	2
	Na_2_B4O_7_ (g·cm^−3^)	-	2
PEO process conditions	Time (s)	360–420	
	Electrolysis voltage (V)	405	
	Average anodic current density (A·dm^−2^)	5	
	Controlled current frequency (Hz)	1000	
	Current rise rate (A·μs^−1^)	200	
	Level of current filling pulse (%)	30	

**Table 3:** Type of samples.

Type of Sample	Designation of Samples
AZ91 magnesium alloy—reference sample	AZ91
AZ91 magnesium alloy covered without the borax addition PEO layer (see Table 2)	Base PEO coating
AZ91 magnesium alloy covered with the borax addition PEO layer (see Table 2)	Modified PEO coating

**Table 4:** Ball-on-Disc test parameters.

Parameter	Value and Unit
Ball material	WC [−]
Ball diameter	6 [mm]
Load	2 [N]
The radius of the sliding circle	6 [mm]
Sliding speed	0.1 [m/s]
Sliding distance	500 [m]
Duration of the test	5 000 [s]
Number of cycles	13 263 [−]

**Table 5:** The EDS analysis of base and modified PEO coatings formed on AZ91 alloy.

Point No. from Figure 4.	Base PEO Coating				Modified PEO Coating			
	O	Mg	Al	Si	O	Mg	Al	Si
1	57.2	31.3	3.1	8.4	50.8	34.6	2.3	12.3
2	48.1	43.7	3.7	4.5	37.2	50.9	6.2	5.7
3	-	91.5	8.5	-	-	91.4	8.6	-
4	-	90.0	10.0	-	-	93.0	7.0	-

**Table 6:** Results of the polarization test of AZ91 magnesium alloy without PEO coating and with coatings manufactured using standard and modified electrolytes.

Polarization Parameter	AZ91	Base PEO Coating	Modified PEO Coating
*E*_corr_ (V)	−1.404	−1.467	−1.494
*i*_corr_ (A cm^−2^)	2.0 × 10^−5^	1.2 × 10^−7^	1.7 × 10^−7^
Rp (Ω·cm^2^)	1297	3.18 × 10^5^	2.58 × 10^5^
corrosion rate (mm/year)	1.396	0.0692	0.0060

## Data Availability

Not applicable.
